# Environmental and social determinants of thyroid cancer: A spatial analysis based on the Geographical Detector

**DOI:** 10.3389/fendo.2022.1052606

**Published:** 2022-11-29

**Authors:** Shirui Huo, Ying Liu, Anyi Sun, Bo Zhang

**Affiliations:** ^1^ School of Life Sciences, Beijing University of Chinese Medicine, Beijing, China; ^2^ School of Chinese Medicine, Beijing University of Chinese Medicine, Beijing, China; ^3^ Department of Ultrasound, China-Japan Friendship Hospital, Beijing, China

**Keywords:** thyroid cancer, environmental, social, risk factor, ecological study, geographical detector

## Abstract

**Introduction:**

Thyroid cancer has increased sharply in China in recent years. This change may be attributable to multiple factors. The current study aimed to explore the environmental and social determinants of thyroid cancer.

**Methods:**

Incidence data from 487 cancer registries in 2016 were collected. Eight factors were considered, namely, air pollution, green space, ambient temperature, ultraviolet radiation, altitude, economic status, healthcare, and education level. A geographical detector (measured by *q* statistic) was used to evaluate the independent and interactive impact of the eight factors on thyroid cancer.

**Results:**

Social factors, especially economic status and healthcare level (*q* > 0.2), were most influential on thyroid cancer.Ultraviolet radiation, air pollution, and temperature had more impact on women, while green space and altitude had more influence on men. Enhanced effects were observed when two factors interacted. Spatially, economic status, healthcare, and air pollution were positively associated with thyroid cancer, while education level, green space, and altitude were negatively related to thyroid cancer.

**Conclusion:**

The socio-environmental determinants and spatial heterogeneity of thyroid cancer were observed in this study. These findings may improve our understanding of thyroid cancer epidemiology and help guide public health interventions.

## Introduction

Thyroid cancer ranks as the 10th most common cancer (1) in the world. Both genetic and environmental factors contribute to the etiology of thyroid cancer. Factors that have been definitively associated with increased risk of thyroid cancer include female sex (female-to-male ratio 3:1); radiation (2, 3); excessive dietary iodine intake (4); mutations of the *RET*, *BRAF*, and *TP53* genes (1); and autoimmune diseases (e.g., Hashimoto’s thyroiditis) (5).

In addition to the traditional factors (e.g., excessive iodine intake), many other environmental factors have been linked to thyroid cancer. These include air pollution (6, 7), green space (8), ambient temperature (9), ultraviolet radiation (10), and geomorphological features(e.g., altitude and slope) (11). A variety of social factors, including economic status, healthcare, and education level (12, 13), have also been associated with thyroid cancer.

A majority of the past studies focused on the impact of a single factor. Also, the geographical area covered by these studies was typically small. The contribution by other socio-environmental factors and the interaction among these factors were neglected. In this study, we took advantage of the vast socio-environmental disparity in China to tackle this complex issue. The current study was based on 487 county-level cancer registries and included 5 environmental and 3 social factors as independent variables in the analysis. Since linear regression analysis could not capture the interaction among the potential risk factors, Geographical Detector (Geodetector) analysis was used as the primary tool (14). The underlying principle of Geodetector analysis is the spatial consistency between the geographical distribution of the target disease and the risk factor. The degree of association is presented as *q* statistic: a value of 1 indicates that incidence variation could be solely explained by the factor, and a value of 0 indicates that there is absolutely no contribution of the factor to incidence variation.

## Materials and methods

### Incidence data

The age-standardized rate (ASR) of thyroid cancer incidence in 2016 was obtained from the 2019 China Cancer Registry Annual Report (the most recent available data). Usually, the cancer incidences of each registry have a 3-year lag behind the current year. This report included a total of 487 subregistries across all 31 provinces, autonomous regions, municipalities, and Xinjiang Production and Construction Corps (15). Particularly, incidence data by gender was used for analysis since the overall incidence of each county was not available. Since all data have been previously published, ethical approval is not applicable.

### Environmental and social data

**Figure 1:**
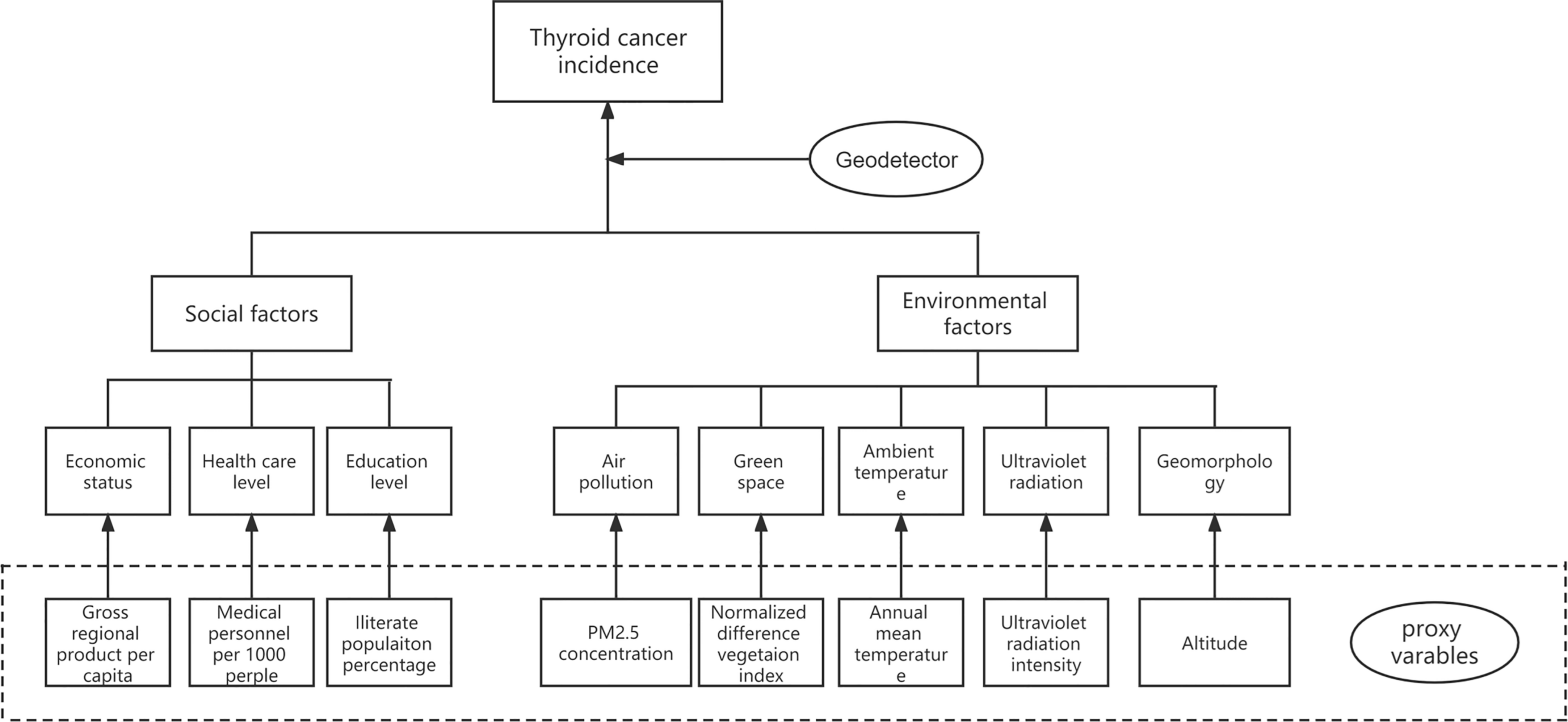
Conceptual framework.

Thyroid cancer determinants from the literature include variant factors in social and environmental aspects, and most of these factors are difficult to measure directly. Nonetheless, the proxy variables of these determinants could be obtained using geographical information system techniques or from published yearbooks (conceptual framework in **Figure 1**).

The analysis included five environmental factors: annual average fine particulate matter concentration (PM2.5), annual average normalized difference vegetation index (NDVI), annual average temperature, annual average ultraviolet radiance, and altitude. These factors were included as potential risk factors based on previous studies and the availability of accurate estimates. Data on PM2.5 (micrograms per cubic meter) were downloaded from NASA Earthdata (16). Annual average NDVI, temperature, and altitude were extracted from the Resource and Environment Science and Data Center (RESDC) (17). Data on ultraviolet radiance were downloaded from the National Earth System Science Data Center (18).

Three social factors were considered at the provincial level: gross regional product (GRP) per capita, medical personnel per 1,000 people (medical level), and illiterate population percentage (education level). GRP per capita and medical personnel per 1,000 people were obtained from China Health Statistics Yearbook 2017 (19). Illiterate population percentage was obtained from China Population and Employment Statistics Yearbook 2017 (20).

### Statistical analysis

Geodetector is based on the core hypothesis that if a risk factor is related to a disease, the distribution of the factor and disease would be spatially consistent (14). This analysis could reduce random effects caused by small samples, and unlike classic linear models, it is immune to collinearity among variables and unrestricted by the assumption of linearity.

The *q* statistic (range: 0–1) is used to measure the effect or determinant power of a certain factor. The *q* statistic is calculated as


$$q=1-\frac{1}{A\sigma^{2}}\sum_{h=1}^{L}\left(A_{h}\cdot \sigma_{h}^{2}\right)$$

In the equation, *A* is the total area of the study region and *σ* ^2^ is the variance of the outcome measure (disease incidence). The incidence is dispersed to the *L* strata (*h* = 1, 2, …*L*). *A_h_* and $\sigma_{h}^{2}$ represent stratum *h* and the variance of stratum *h*. *q* = 1 indicates that the variation in disease incidence can be explained completely by a given factor. *q* = 0 indicates that there is no association between disease incidence and the factor. Statistical significance was set at *P*<0.05 (tested using the Geodetector software).

Three detectors of this statistical tool that were implemented in the current study are as follows:

1) The factor detector quantifies the determinant power of each explanatory factor.
2) The interactive detector reveals if two factors, *x*_1_ and *x*_2_, have an interactive impact on thyroid cancer. This relationship is quantified by

$q(x_{1}\cap x_{2})$ including five intervals as follows: weaken, non-linear,

$q(x_{1}\cap x_{2})<Min(q(x_{1}),q(x_{2}))$

$Min(q(x_{1}),q(x_{2}))<q(x_{1}\cap x_{2})<Max(q(x_{1}),q(x_{2}))$

$q(x_{1}\cap x_{2})>Max(q(x_{1}),q(x_{2}))$

$q(x_{1}\cap x_{2})=q(x_{1})+q(x_{2})$

$q(x_{1}\cap x_{2})>q(x_{1})+q(x_{2})$

*Min*(*q*(*x*_1_),*q*(*x*_2_)),*Max*(*q*(*x*_1_),*q*(*x*_2_)) refer to the minimum and maximum *q* values of two detected factors, respectively.
3) The risk detector maps statistically significant differences in thyroid cancer incidence in the categories of an explored factor.

Since the analysis is more reliable when exposures are included as categorical data (21), continuous variables were transformed into categorical variables, as previously described (22). The stratification results are shown in **Table 1**. All data were processed and extracted in grids (10 km × 10 km) with a fishnet tool in ArcGIS 10.7 and then analyzed in Excel format by Geodetector.

**Table 1:** Classification results and strategies of nine factors.

Factor	Stratification intervals							Stratification method
	1	2	3	4	5	6	7	
Per capita GRP (yuan)	27,643–35,184	35,184–40,432	40,432–40,564	40,564–47,194	47,194–72,064	72,064–118,198		Quantile
Medical level (1/1,000 people)	4.5–5.2	5.2–6	6–6.3	6.3–6.8	6.8–7.1	7.1–10.8		Quantile
Education level (%)	1.56–3.6	3.6–3.79	3.79–4.83	4.83–8.22	8.22–13.45	13.45–41.12		Quantile
PM2.5 (μg/m^3^)	2.05–4.1	4.1–5.97	5.97–8.04	8.04–13.8	13.8–23.91	23.91–35.55	35.55–93.85	Natural breaking
NDVI	0.07–0.19	0.19–0.35	0.35–0.53	0.53–0.66	0.66–0.75	0.75–0.82	0.82–0.89	Natural breaking
Temperature (°C)	<0	0–5	5–10	10–15	15–20	>20		According to RESDC
Ultraviolet (MJ/m^2^)	0.67–0.82	0.82–0.93	0.93–1.02	1.02–1.11	1.11–1.2	1.2–1.33	1.33–1.5	Natural breaking
Altitude (m)	0.01–355.7	355.7–809.4	809.4–1,259.81	1,259.81–1,963.19	1,963.19–3,083.3	3,083.3–4,263.49	4,263.49–5,147.62	Natural breaking

GRP, gross regional product per capita; MED, medical level: medical personnel per 1,000 people; education level: illiterate population percentage; PM2.5, fine particulate matter; NDVI, normalized difference vegetation index; RESDC, Resource and Environment Science Data Center (https://www.resdc.cn/).

## Results

### Thyroid cancer incidence in China in 2016

**Figure 2:**
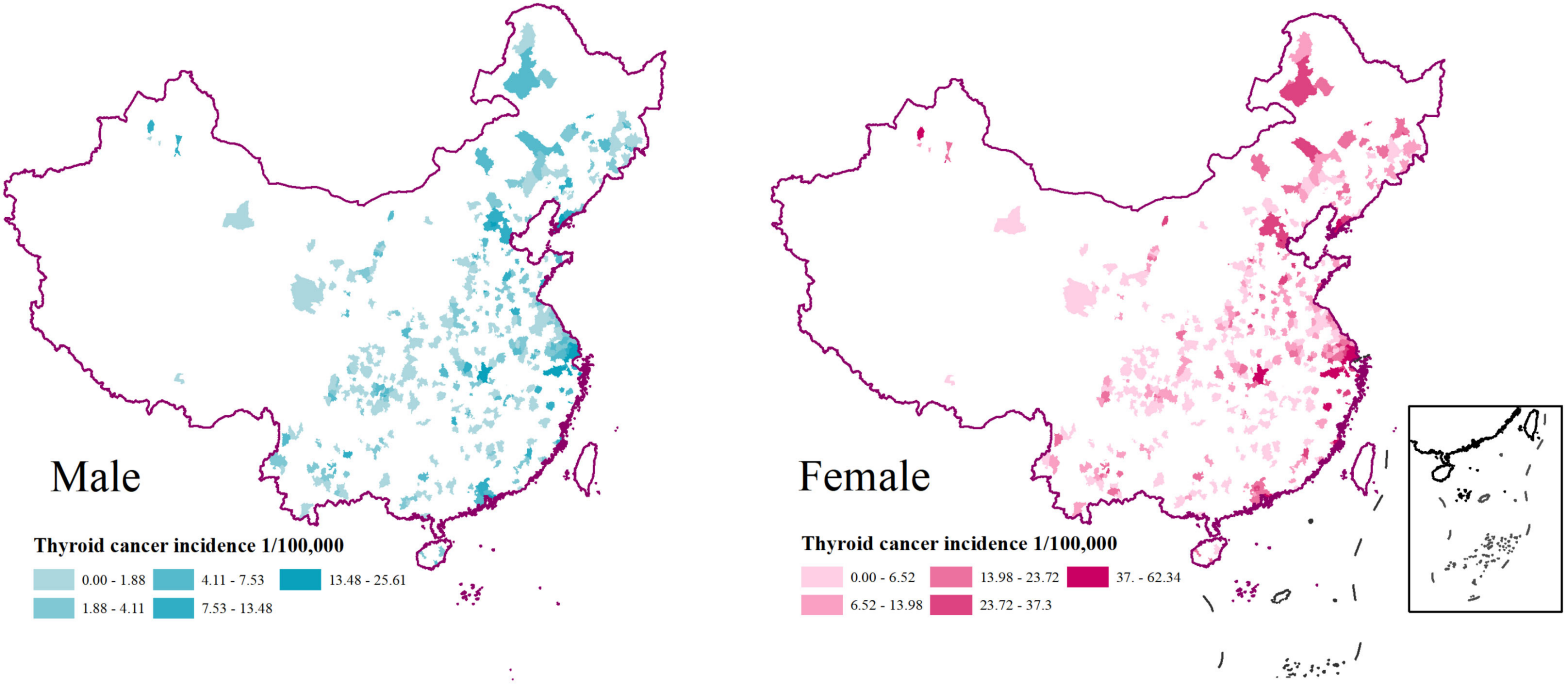
Spatial distributions of 487 registries and TC incidence by sex in China, 2016.

In 2016, 487 cancer registries were included, covering a total of 382 million subjects (194 million men, 188 million women), about 27.60% of the total population in China. Overall, there were 50,424 new thyroid cancer cases (12,240 men and 38,184 women). The overall ASR of thyroid cancer incidence was 9.70/10^5^ with 4.71/10^5^ in men and 14.79/10^5^ in women (15). The spatial distributions of thyroid cancer incidence by gender are shown in **Figure 2**. Overall, thyroid cancer incidence in women was about 3 times higher than that in men. Higher incidence rates were observed in eastern areas.

### Environmental and social determinants of thyroid cancer

**Figure 3:**
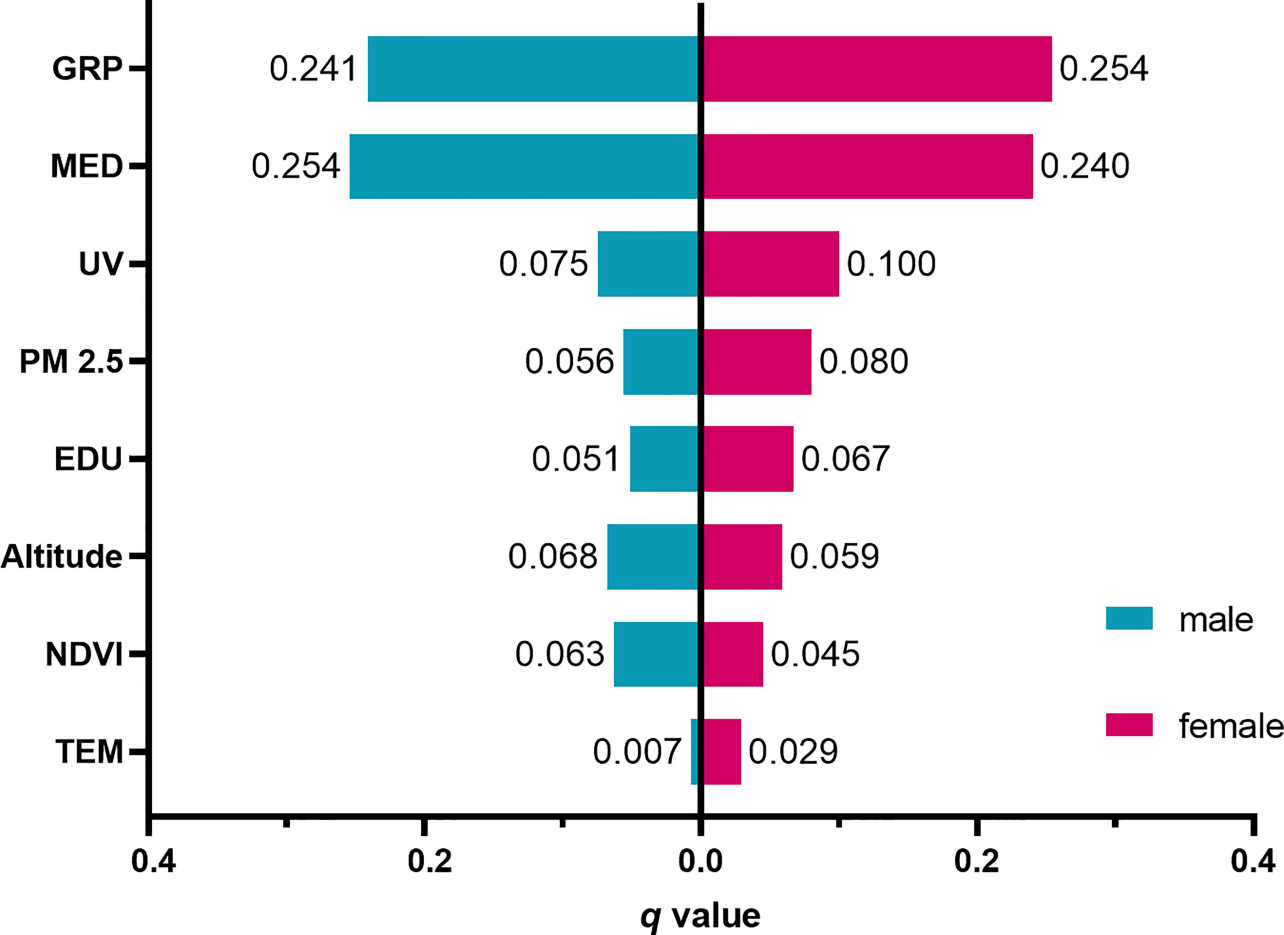
Results of the factor detector by gender. GRP, gross regional product per capita; MED, medical level; UV, ultraviolet radiation; PM2.5, fine particulate matter; EDU, education level; NDVI, normalized difference vegetation index; TEM, temperature.

The results of the factor detector are shown in **Figure 3**. All factors had statistically significant determinant power on thyroid cancer (*P*< 0.05). According to their *q* values, GRP was the most influential factor that determined the thyroid cancer distribution in women, followed by medical level, ultraviolet radiation, PM2.5, education level, altitude, NDVI, and temperature. As for men, medical level was the most influential factor of thyroid cancer, followed by GRP, ultraviolet radiation, altitude, NDVI, PM2.5, education level, and temperature. For both genders, GRP (men: 0.241, women: 0.254) and MED (men: 0.254, women: 0.240) had the largest impact on thyroid cancer. Most environmental factors had smaller (*q*< 0.1) determinant power especially temperature which had a *q* value of only 0.007 in men which implied that this factor had a little influence on men. Noticeably, ultraviolet radiation (*q* value in women: 0.100) and PM2.5 (*q* value in women: 0.080) ranked as the third and fourth most dominant factors in women. Another gender difference that was found was that most environmental factors had more determinant power on women, except altitude (men: 0.068, women: 0.059) and NDVI (men: 0.063, women: 0.045).

### Interactions between paired factors

**Figure 4:**
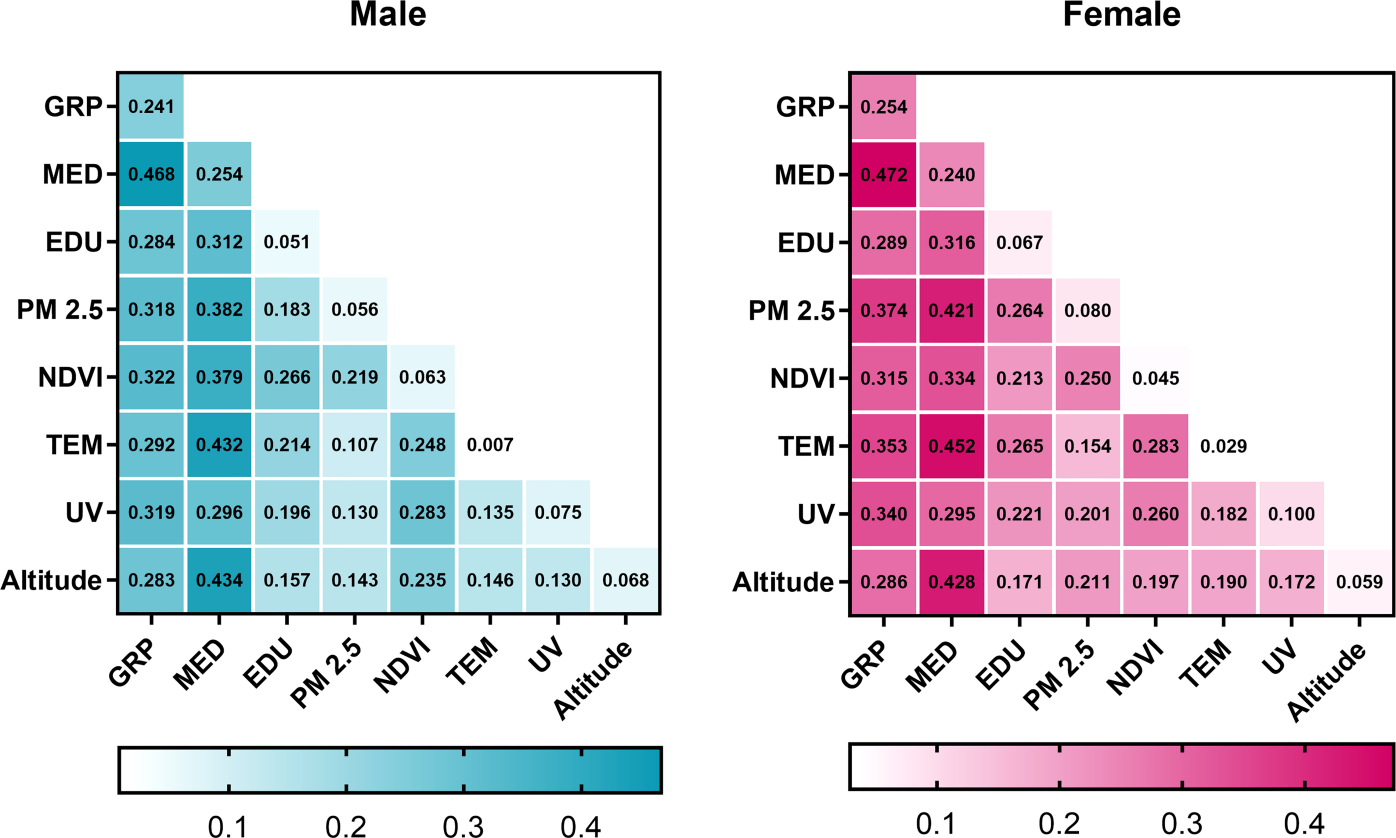
Interactive results between paired factors, by sex. Green and magenta colors represent men and women, respectively. The diagonal squares were labeled with independent *q* value of each factor. The intersection of each row and each column represents the interactive determinant power of paired factors. GRP, gross regional product per capita; MED, medical level; UV, ultraviolet radiation; PM2.5, fine particulate matter; EDU, education level; NDVI, normalized difference vegetation index; TEM, temperature.

The interactive detector was implemented to find out if two potential risk factors worked separately or independently. The outcomes are presented in **Figure 4**. Interactions of any paired factors could enhance their impact on thyroid cancer, either a bivariate enhancement or a non-linear one. To interpret the results, the interaction of two pairs of factors including GRP and medical level and GRP and PM2.5 were used as examples. The independent determinant power of GRP, medical level, and PM2.5 on men were 0.241, 0.254, and 0.056, respectively. However, the combination of GRP and medical level enhanced each other with a *q* value of 0.468 (less than their sum of 0.495 but bigger than either of their independent *q*), representing a bivariate enhancement. This also indicated that though the enhanced interaction was compromised, medical level was more dominant since its *q* value is closer to the sum. GRP and PM2.5 operated together resulting in a non-linear enhancement with a *q* value of 0.318 (bigger than their sum of 0.297), which implied that PM2.5 and GDP immensely strengthened the determinant power of each other.

### Spatial patterns of thyroid cancer

**Figure 5:**
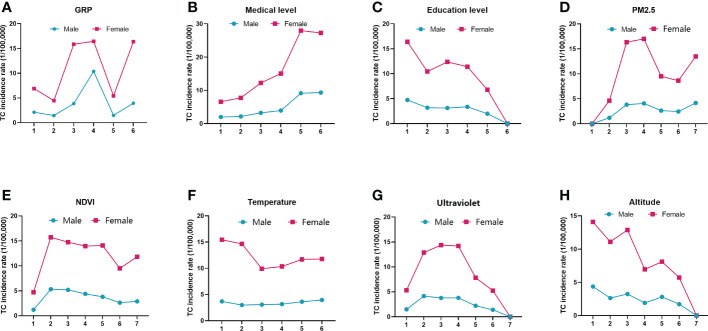
Spatial distributions of thyroid cancer incidence rates in different intervals of eight factors. The intervals of each factor are given along the horizontal axis. The vertical axis represents the thyroid cancer incidence rate (1/10^5^). Solid dots and squares are the average thyroid cancer incidence rates in the different subtypes of the eight factors. Green and magenta colors represent men and women, respectively. **(A)** GRP, gross regional product; **(B)** medical level; **(C)** education level; **(D)** PM2.5, fine particulate matter; **(E)** NDVI, normalized difference vegetation index; **(F)** temperature; **(G)** ultraviolet; **(H)** altitude.

The results of the risk detector are shown in **Figure 5**. Thyroid cancer incidence rates were positively related to PM2.5 concentrations. In the areas with PM2.5 concentration higher than 5.97 μg/m^3^, the thyroid cancer incidence was significantly higher than that in areas with PM2.5 concentration lower than 5.97 μg/m^3^. In registries with ultraviolet radiation between 0.67 and 1.11 MJ/m^2^, thyroid cancer incidence increased; however, it declined as the radiation increased to more than 1.11 MJ/m^2^. Noticeably, thyroid cancer incidence rates in women declined with increasing temperature and altitude. The incidence in areas with a temperature lower than 5°C was 50% higher than that in warmer areas. It is also worth noting that the negative association between NDVI and thyroid cancer incidence rates was more evident in men than in women. In areas with NDVI between 0.19 and 0.35, the incidence in men was about 2 times higher than in areas with NDVI between 0.82 and 0.89.

For social factors, thyroid cancer incidence rates were positively related to GRP and medical level but negatively related to education level. The incidence rates in regions with the highest number of medical personnel (7.1–10.8 per 1,000 people) were nearly 4 times (for women) and 5 times (for men) higher than those in regions with medical personnel between 4.5 and 5.2 per 1,000 people. Though larger fluctuations of thyroid cancer incidence rates were shown when GRP or education level increased, higher incidence rates were often found in regions with better economic status.

## Discussion

To the best of our knowledge, this Geodetector-based study is novel in terms of exploring multiple socio-environmental determinants of thyroid cancer on a large spatial scale in China. In addition, our study was under ecological design and collected data from a publicly available data source, which provided an economical and easier way to help develop a better understanding of thyroid cancer epidemiology.

Our results revealed that social factors especially medical level and economic status were the most dominant determinants (*q* > 0.2) of thyroid cancer in China in 2016. Moreover, thyroid cancer incidence rates appeared to be higher in areas with better development (higher per capita GRP, medical level, lower illiterate rate), which indicated that individuals in these areas had more access to healthcare with good quality; thus, potential overdiagnosis trend might have existed in China. These findings are consistent with previous research. Studies conducted in the USA and Australia (23, 24) found that more access to medical resources and the application of new diagnostic practices might be partially responsible for the rising thyroid cancer incidence, especially papillary thyroid cancer. One Chinese population-based study (25) estimated that in 2017, approximately 1,000 individuals might have been overdiagnosed with thyroid cancer daily, and during the period 2019–2030, more than 5 million patients could be overdiagnosed with thyroid cancer. Therefore, interventions are urgently needed to reduce the overtreatment of thyroid cancer, such as inventing more accurate diagnostic tools or guidelines.

The results of the factor detector showed that most environmental factors had less impact (*q*< 0.1) on thyroid cancer, but the risk detector disclosed that thyroid cancer incidence tended to increase in registries with severe ambient pollution, less green space, cold climate, more ultraviolet radiation, and lower altitude. Though the basis of the correlations between these environmental factors and thyroid cancer is not well-established, there are some possible explanations. PM2.5 is a surrogate parameter for air pollution and has been classified as one of the carcinogens by the International Agency for Research on Cancer (IACR) (26). It contains non-metal oxides and heavy metals, which could induce oxidative stress reaction leading to DNA damage (27, 28). Additionally, a complex of compounds in PM2.5 could disturb thyroid functions (29) which might potentially increase the risk of thyroid autoimmune diseases that are known as thyroid cancer-related (5). As for cold climate, an inverse correlation between thyroid cancer and temperature has been described in a previous study conducted in the USA (9). Our study observed that thyroid cancer incidence in women was higher in areas with lower ambient temperature in China though temperature had a very limited impact on women with a determinant power of 0.029. The possible bases for this finding are as follows: first, thyroid-stimulating hormone (TSH) increases with the decrease of environmental temperature (29); second, the inherent difference of thyroid hormone level between women and men (30) and a higher level of TSH were defined as a risk factor in some studies (31). Therefore, people, especially women, living in a cold environment are more likely to keep a higher TSH level than women living in a warmer climate and become more prone to thyroid cancer.

NDVI, a parameter implicating vegetation coverage, is often associated with insect-borne, tropical diseases (32). Some investigations mentioned that green space might be protective against several cancers, consistent with our findings that thyroid cancer incidence declined with the increase of vegetation coverage (33, 34) except in regions with poor vegetation and fewer cancer registries since these areas are not ideal habitats. Even though there is no well-established theory to explain the current findings, we speculate that higher vegetation coverage may enhance air quality; thus, exposure to harmful chemical compounds could be reduced.

To date, the results from previous studies suggested that variations in thyroid hormones might be correlated to the change in meteorological factors, but the results were not conclusive (35). In our study, we found that ultraviolet radiation had a certain influence on thyroid cancer (*q*< 0.1). There was a non-linear relationship between thyroid cancer and ultraviolet radiance. Our results showed that the annual average of ultraviolet radiation in northern, northwestern, and southeastern China exceeded 0.93 MJ/m^2^; thus, individuals living in these regions are exposed to more ultraviolet radiation. Ultraviolet radiation is considered to induce DNA damage, leading to skin cancer (36). A prospective study in France reported that residential UV exposure is associated with the risk of thyroid cancer (37). Higher thyroid cancer incidence rates observed in these areas might be partially due to increased ultraviolet radiation exposure. Noticeably, we also observed that as the ultraviolet radiation increased to 1.11 MJ/m^2^, the thyroid cancer incidence declined. Though there is no well-established theory for this trend, our explanation is that, due to the health and economic disparities between eastern and western China (38), only a few cancer registries have been established in areas with ultraviolet radiation exposure at more than 1.11 MJ/m^2^ (mainly in northwestern and southwestern China). Therefore, a small sample size might not reflect the true effect of exposure to higher ultraviolet radiation.

Another possible explanation is that UV-induced gene damage could be repaired by certain proteins such as DNA photolyase/cryptochrome family, methyltransferase-like 14 (37, 39). It is possible to say that residents habituated to a high level of ultraviolet radiation may have a greater resistance to UV-induced DNA damage by developing a stronger DNA repair response.

One study in the USA also reported a non-linear relation between ultraviolet radiation exposure and thyroid cancer (10); however, a higher ultraviolet radiation exposure increased the risk of thyroid cancer. As for ELE, higher thyroid cancer incidences were observed in low-altitude areas. A possible basis for these findings is that other socio-environmental features on different elevations such as the economic development level, endocrine-disturbing chemicals (EDCs) in the soil and air pollution might also play important roles in the development of thyroid cancer (40). As previous studies suggested (11, 41), on the one hand, human activities, especially industrial production and transportation, tend to take place in lower and flat lands, which could discharge a tremendous amount of deleterious chemicals; on the other hand, environmental pollutants are more likely to accumulate in low-altitude habitats (21). It is plausible to assume that people living in lower lands have much higher exposure to harmful chemicals through the food chain and respiratory pathway, which could increase the risk of thyroid cancer.

It is also worth noting that the interaction between any paired environmental and social factors enhanced each other’s impact on thyroid cancer and the risk areas of all factors were also detected. Taken together, the interests in further research and prevention strategies for thyroid cancer should vary according to the social and environmental characteristics of the study areas. For example, in northern and eastern China, where the health resources are concentrated and air pollution is a challenge, future thyroid cancer epidemiology investigations should focus on overdiagnosis and ambient pollution. In addition, in high-latitude areas such as northwestern China and inner Mongolia, the risk of ultraviolet radiation and cold climate should be taken into consideration.

There are limitations to our study. One of them is ecological fallacy, a common and inherent flaw of ecological studies since the inferences about the nature of individuals are based on the population to which they belong (42). Consequently, our results may not be able to reveal the true relationship between risk factors and thyroid cancer. Individual-level studies should be carried out in the future. Moreover, gender was the only confounding factor considered since other baseline information on population such as age distribution of each registry was not available. We would like to include more confounding factors in our further studies. Another problem is that thyroid cancer includes various subtypes (e.g., papillary, follicular, medullar, and anaplastic), but the environmental or social risk factors for these subtypes remain unclear. The calculation of ASR of thyroid cancer, according to the 2019 China Cancer Registry Annual Report, was based on the new cases from all subtypes of thyroid cancer. However, the incidence of each thyroid cancer subtype was not mentioned in the published data source. Although we have tried to obtain the incidence data of thyroid cancer subtypes, to date, the available data were not found. Perhaps this problem could be addressed in our future work. Lastly, though the incidence data of 2016 with a bigger sample size enabled us to explore the spatial patterns of thyroid cancer with more precision, this study was under a cross-sectional design, which could not reflect the temporal trends of thyroid cancer determinants.

## Conclusions

The socio-environmental determinants of thyroid cancer and spatial heterogeneity of thyroid cancer were observed in this study. Social factors, especially economic status and healthcare, exhibited more influences on thyroid cancer, suggesting potential overdiagnosis trends. Interactions between any two factors enhanced their impact on thyroid cancer. Spatial heterogeneity of thyroid cancer indicated that ambient pollution, less green space, cold climate, and ultraviolet radiation were linked to higher thyroid cancer incidences. These findings may help guide public health interventions and improve our understanding of thyroid cancer epidemiology in China. However, this study is under an ecological design, and future investigations at the individual level are needed to confirm our findings.

## Data availability statement

The raw data supporting the conclusions of this article will be made available by the authors, without undue reservation.

## Ethics statement

Ethical review and approval was not required for the study on human participants in accordance with the local legislation and institutional requirements. Written informed consent from the participants’ legal guardian/next of kin was not required to participate in this study in accordance with the national legislation and the institutional requirements.

## Author contributions

SH and BZ contributed to the conceptualization of the study. SH, YL, and BZ completed the methodology. SH applied the software. SH and AS contributed to the data curation and formal analysis. BZ and YL contributed to the investigation. BZ and YL provided the resources. SH wrote the original draft. BZ and YL were responsible for the review and editing of the manuscript, supervision, and project administration. BZ was responsible for the funding acquisition. All authors have read and agreed to the published version of the manuscript.

## Funding

This research was funded by the Beijing Municipal Natural Science Foundation (Grant No. 7192152) and the Discipline Construction Project of Peking Union Medical College (Grant No. 201920102305).

## Acknowledgments

The authors thank Dr. L. Wang for the valuable advice on statistical analyses and K. Zhang for the text review and valuable advice.

## Conflict of interest

The authors declare that the research was conducted in the absence of any commercial or financial relationships that could be construed as a potential conflict of interest.

## Publisher’s note

All claims expressed in this article are solely those of the authors and do not necessarily represent those of their affiliated organizations, or those of the publisher, the editors and the reviewers. Any product that may be evaluated in this article, or claim that may be made by its manufacturer, is not guaranteed or endorsed by the publisher.
